# Urinary Bladder Carcinoma in Females: A Clinico-Pathological Assessment

**DOI:** 10.7759/cureus.39753

**Published:** 2023-05-30

**Authors:** Rahul Gupta, Suhail M Khan, Manik Mahajan, Poonam Sharma, Arti Mahajan

**Affiliations:** 1 Urology, Government Medical College, Jammu, Jammu, IND; 2 Radiology, Government Medical College, Jammu, Jammu, IND; 3 Pathology, All India Institute of Medical Sciences Vijaypur, Jammu, IND; 4 Anaesthesia, Government Medical College, Jammu, Jammu, IND

**Keywords:** transitional cell carcinoma, hematuria, female, malignancy, urinary bladder carcinoma

## Abstract

Background: Urinary bladder cancer is an uncommon cancer in females. Despite not being an infrequent encounter, female bladder cancer remains a poorly defined entity. There is a paucity of literature regarding bladder cancer in females, especially in North India.

Objective: This study aims to evaluate the clinico-pathological profile of bladder cancer in female patients managed at a single centre in north India.

Materials and methods: This retrospective observational study was carried out in a tertiary care centre in North India. Medical records and a database of female patients with bladder cancer treated between January 2012 and January 2021 were retrieved. Data regarding age, duration of disease, associated comorbidity, histopathological variants, and outcomes were studied.

Results: Out of 56 female patients with bladder masses, 55 had transitional cell carcinoma (TCC), while one had pheochromocytoma. Painless hematuria (80.3%) was the commonest presentation. At the time of presentation, 5 patients (9.1%) had muscle-invasive bladder cancer (T2-T4), while 50 patients had non-muscle-invasive disease, out of which 31 (56.4%) patients had high-grade and 19 (34.5%) patients had low-grade papillary carcinoma. Twenty-three patients (41.8%) had a history of exposure to domestic *chulha* (open wood-burning cooking stove), and 11 patients (20%) were smokers; six patients (10.9%) were exposed to both risk factors.

Conclusions: Female bladder cancer was most prevalent in the sixth decade of life, with the majority of patients having a high-grade but non-muscle-invasive disease. Of all the risk factors, *chulha* exposure was the main risk factor in the aetiology of female bladder cancer.

## Introduction

Urinary bladder cancer is the sixth-commonest malignancy in the world and the second most common urological malignancy, preceded only by carcinoma of the prostate [1]. It is three to four times more prevalent in men than in women [2]. The overall annual incidence of urinary bladder cancer is 2.4% among males and 0.7% among females [3]. Bladder cancer is one of the leading causes of cancer-related mortality in developed countries and accounts for 3.4% of the global cancer burden [4].

The most common histological variant is transitional cell carcinoma (TCC), accounting for 90% of all cases [5]. The risk factors for bladder cancer include exposure to chemicals, tobacco consumption, and urinary schistosomiasis. In the general population, age, sex, and race are key determinants of survival outcomes and prognosis.

According to the literature, women usually present with more advanced diseases, leading to poorer prognoses [6]. There is a paucity of literature on bladder carcinoma in females, especially in the Indian context. This study aimed to evaluate the clinicopathological characteristics of the disease in the female population in our region.

## Materials and methods

Study design and setting

This hospital-based retrospective descriptive study was carried out in a tertiary care hospital in North India, which caters to both rural and urban populations. Ethical clearance was obtained from the ethical committee of the institute (IEC/GMC/Cat C/2021/493).

Study population

We retrospectively evaluated patients with bladder carcinoma who were managed in a tertiary care hospital in North India over a period of nine years (from January 2012 to January 2021). Of these, female patients with newly diagnosed bladder carcinoma were included in this study. Patients who had received primary treatment at other hospitals, patients with recurrence, or patients with non-malignant lesions on histopathology were excluded.

Methods

A comprehensive data assessment was done for all enrolled patients. Data retrieved included a detailed history (duration of illness), baseline investigations (radiological imaging, cystopan endoscopy [CPE]), and per-operative and histopathological findings.

Surgical management

All patients with bladder masses were subjected to monopolar trans-urethral resection of bladder tumours (TURBT) under general anaesthesia using a 22 Fr resectoscope after obtaining clearance from the anaesthesia department. Further management was planned according to the histopathology. Patients with the high-grade muscle-invasive disease were subjected to laparoscopic radical cystectomy with anterior pelvic exenteration, hysterectomy with bilateral salpingectomy ± oophorectomy, and excision of the anterior 1/3 of the vagina with an ileal conduit. Those with the low-grade non-muscle invasive disease were subjected to Bacillus Calmette-Guerin (BCG) instillations as per Lam's protocol. Mitomycin 40 mg was reserved for those who developed BCG cystitis or did not tolerate BCG. Indwell time for intravesical instillation was two hours. These patients were followed up every three months for two years and annually thereafter if no recurrence was reported.

Statistical analysis

All categorical data were entered in MS Excel format (Microsoft® Corp., Redmond, WA), expressed as numbers and proportions, and presented in tables.

## Results

During the study period, a total of 612 patients were admitted to the hospital with bladder masses. Of these, 56 (9.15%) patients were females, while the rest were males (approximate male/female ratio 10:1). Among the 56 women, one woman was found to have pheochromocytoma of the urinary bladder on histopathological examination and was thus excluded from the study. Therefore, a total of 55 women with bladder cancer (mean age at presentation: 56.9 years [range, 25-82]) were included in this study. The age distribution of patients is as follows: 60-79 years, 43%; 40-59 years, 31%; 20-39 years, 15%; and >80 years, 11% (Table 1). Thus, a vast majority of patients (76.3%) were in the post-menopausal age group.

**Table 1:** Age distribution of patients with bladder cancer (n=55)

Age group (in years)	Number (%)
20–39 years	08 (15%)
40–59 years	17 (31%)
60–79 years	24 (43%)
>80 years	06 (11%)
Total	55 (100%)

Eleven patients (20%) had a history of cigarette/tobacco smoking, while 23 patients (41.8%) had exposure to chulha while cooking. Six patients (10.9%) had a history of both cigarette smoking as well as chulha exposure, while 27.3% had no significant exposure to predisposing factors. Out of 55 patients, diabetes melitus was present in 12 patients (21.8%), while hypertension was observed in 8 patients (14.5%).

The clinical presentations are presented in Table 2. Painless gross hematuria was the commonest presenting symptom (45 [81.8%] patients). The average time elapsed between the onset of symptoms and presentation was 4.3±1 weeks.

**Table 2:** Symptoms at presentation (n=55) LUTS: lower urinary tract symptoms

Presenting symptoms	Number (%)
Hematuria	45 (81.8%)
Pain/poor urine flow	04 (7.3%)
Flank pain	01 (1.8%)
LUTS + hematuria	10 (18.2%)
LUTS	08 (14.5%)

**Figure 1:**
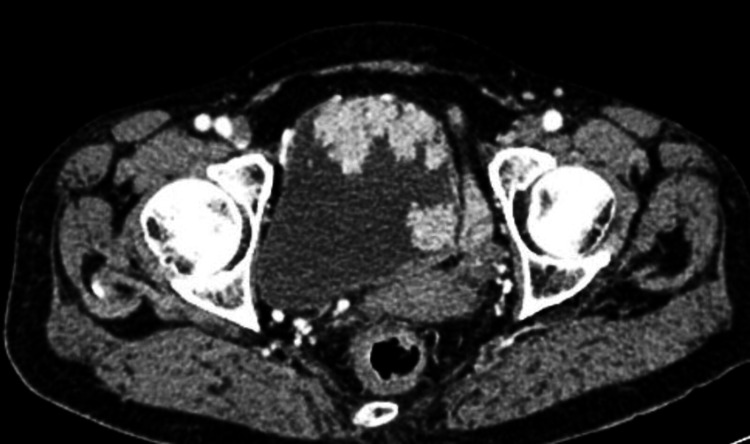
Multiple bladder masses Axial contrast-enhanced computed tomographic image showing multiple enhancing bladder masses

The tumour distribution on CPE is presented in Table 3. All patients in this cohort had transitional cell carcinoma. Five patients (9.1%) had muscle-invasive bladder cancer (high-grade) (Figure 1); 50 patients had non-muscle-invasive disease, out of which 31 (56.4%) patients had high-grade and 19 (34.5%) patients had low-grade papillary carcinoma invading lamina propria.

**Table 3:** Findings on cysto-pan endoscopy (n=55)

Site of tumor	Number
Left lateral wall	21
Right lateral wall	14
Posterior wall	6
Multiple masses	14

On follow-up, 10 patients (20.0%) developed recurrence within two years; of these, seven patients (14.0%) had recurrence within one year, and three patients (6.0%) developed recurrence in the second year. Recurrence was documented on routine CPE follow-up visits. All patients with recurrence were subjected to re-resection and were found to have low-grade disease. None of the recurrences were at the primary site of resection.

## Discussion

Females with bladder cancer tend to present with more advanced disease compared to men [7-10]. Although not an uncommon entity, the literature on female bladder cancer is quite sparse. In this study, we sought to characterize the clinicopathological characteristics of bladder cancer in females. Some of our findings were contrary to those reported in the literature. The male-to-female ratio among bladder cancer patients in our setting (10:1) was much greater than that reported in the literature (3:1) [2]. This difference may be attributed to bias related to social, economic, and educational factors. Another possible reason may be reduced exposure to industrial carcinogens/chemicals, as the majority of the females in our region were homemakers.

Exposure to cigarette smoking is the most relevant risk factor for urinary bladder carcinoma. Irrespective of sex, smoking is associated with up to a six-fold higher risk of urinary bladder carcinoma [11,12]. This is attributed to the secretion of alpha and beta naphthylamine in the urine of smokers, which is a recognized causative factor [13]. In bladder carcinoma, smoking has a dose-dependent detrimental effect on survival [14,15]. In our series, 11 patients (20%) were smokers, 23 patients (41.8%) had exposure to chulha cooking, and six patients (10.9%) had a history of exposure to both. This may be attributed to the social strata of the female patients who were operated on at our centre.

The most common presenting symptom of bladder cancer is painless hematuria or storage voiding symptoms [16]. In our study too, hematuria alone or hematuria with lower urinary tract symptoms was the initial presenting symptom in 80.3% and 18.1% of patients, respectively. We hypothesize that the sight of blood in the urine may prompt a patient to seek medical advice earlier than they would for other symptoms. Moreover, storage symptoms can delay the diagnosis in female patients, as these are difficult to differentiate from symptoms of urinary tract infection, especially in post-menopausal females. Moreover, these patients tend to present late as they typically receive empirical treatment with antibiotics, which affords temporary symptomatic relief [17].

Transitional cell carcinoma constitutes almost 90% of bladder cancer cases, according to published literature [18]. All the patients in this study had transitional cell carcinoma, except for one patient who had pheochromocytoma. At the time of presentation, 25-29% of female patients with bladder cancer had muscle-invasive disease [17,18]. Moreover, female patients tend to have a higher incidence of high-grade, multiple, and larger tumours at the time of presentation [19,20]. In contrast, although 56.3% (31/55) of the patients in our study had high-grade malignancy, only 9.1% of patients had the muscle-invasive disease at presentation.

Patients with non-muscle-invasive diseases have a high overall survival rate. However, 60-70% of non-muscle-invasive tumours recur, and 5-25% of pTa and pT1 tumours advance to muscle-invasive cancer [18]. The multiplicity of the tumour is the most important factor in determining recurrence risk, followed by tumour volume, grade, and T subgroup. In our series, 14 (25.5%) patients had multiple bladder lesions.

Bladder cancer in females is thought to be more aggressive and of a higher grade at initial diagnosis. This is attributed to the late presentation and diagnosis. Our findings were contradictory to those in previous reports. In our study, the majority of patients (81.8%) fared well and showed no signs of recurrence at two-year follow-up. Seven patients (12.7%) had recurrence within one year, and three patients (5.5%) in the second year. These patients were asymptomatic and were detected on follow-up CPE. All were subjected to resection and had low-grade disease. None of the recurrences were at the primary site of resection.

Our study had a few limitations. First, it was a retrospective study. Second, immunohistochemistry of the specimens was not available/carried out to stratify muscle-invasive bladder cancer into different subtypes, as this low-cost technique helps in predicting prognosis.

## Conclusions

Urinary bladder cancer is less common in females as compared to males, but if detected in time, it is associated with a good prognosis. The commonest age at presentation in our study was the sixth decade of life, with the majority having a high-grade but non-invasive transitional cell carcinoma. Chulha exposure in rural areas appears to be a possible risk factor in the development of bladder cancer in females.
